# Towards Gene Banking Amphibian Maternal Germ Lines: Short-Term Incubation, Cryoprotectant Tolerance and Cryopreservation of Embryonic Cells of the Frog, *Limnodynastes peronii*

**DOI:** 10.1371/journal.pone.0060760

**Published:** 2013-04-05

**Authors:** Bianca Lawson, Simon Clulow, Michael J. Mahony, John Clulow

**Affiliations:** School of Environmental and Life Sciences, University of Newcastle, Callaghan, New South Wales, Australia; Federal University of Parana (UFPR) – Campus Palotina, Brazil

## Abstract

Gene banking is arguably the best method available to prevent the loss of genetic diversity caused by declines in wild populations, when the causes of decline cannot be halted or reversed. For one of the most impacted vertebrate groups, the amphibians, gene banking technologies have advanced considerably, and gametes from the male line can be banked successfully for many species. However, cryopreserving the female germ line remains challenging, with attempts at cryopreserving oocytes unsuccessful due to their large size and yolk content. One possible solution is to target cryopreservation of early embryos that contain the maternal germ line, but consist of smaller cells. Here, we investigate the short term incubation, cryoprotectant tolerance, and cryopreservation of dissociated early embryonic cells from gastrulae and neurulae of the Striped Marsh Frog, *Limnodynastes peronii*. Embryos were dissociated and cells were incubated for up to 24 hours in various media. Viability of both gastrula and neurula cells remained high (means up to 40–60%) over 24 hours of incubation in all media, although viability was maintained at a higher level in Ca^2+^-free Simplified Amphibian Ringer; low speed centrifugation did not reduce cell viability. Tolerance of dissociated embryonic cells was tested for two cryoprotectants, glycerol and dimethyl sulphoxide; dissociated cells of both gastrulae and neurulae were highly tolerant to both—indeed, cell viability over 24 hours was higher in media containing low-to-medium concentrations than in equivalent cryoprotectant-free media. Viability over 24 hours was lower in concentrations of cryoprotectant higher than 10%. Live cells were recovered following cryopreservation of both gastrula and neurula cells, but only at low rates. Optimal cryodiluents were identified for gastrula and neurula cells. This is the first report of a slow cooling protocol for cryopreservation of amphibian embryonic cells, and sets future research directions for cryopreserving amphibian maternal germ lines.

## Introduction

There is a growing interest in cryobanking amphibian cells and tissues as a conservation tool [1]–[6] to address the catastrophic collapse in amphibian biodiversity that has been well documented since 1990 [7]–[13]. The most obvious target for cryopreservation is the embryo; however, this is not likely to be achieved in the near term, given the failure of progress in cryopreserving fish oocytes and embryos, which have similar structural and biochemical properties such as large cell size and high yolk composition [14]–[18]. This is unfortunate, as external fertilisation and development is the norm in anurans, and the resumption of development of cryopreserved embryos would complete a simple procedure to store, retrieve and restore the diploid genome (mitochondrial and nuclear) without the need for additional complicated post thaw procedures such as implantation. The cryopreservation of embryonic cells is an alternative, but more circuitous path, to storing and retrieving the diploid genome, and will require the reconstitution of embryos through either nuclear transfer or the generation of chimeras. These procedures have been documented for various fish species with reports of the cryopreservation of blastomeres from dissociated embryos [19]–[27] and the generation of chimeras from unfrozen [28]–[31] and cryopreserved blastomeres [32]–[35]. There is also one report of the generation of nuclear transfer embryos in an anuran species from cryopreserved blastomeres [36].

Despite the approach being investigated for fish, other than the single report above, there are no reports of the cryopreservation of early embryonic cells from anuran amphibians.

This is despite the existence of a large literature and long history of nuclear transfer in amphibians [37]. The development of procedures for the isolation and cryopreservation of embryonic cells is a necessary step towards the routine generation of nuclear transfer or chimeric embryos from cryopreserved anuran embryonic cells. This paper reports a study of the isolation, incubation, tolerance to cryoprotectants and cryopreservation of cells from early embryos of the frog, *Limnodynastes peronii* (the Striped Marsh Frog, a common temperate species of south east Australia). The recovery of live cells from gastrulae and neurulae, following cryopreservation with the penetrating cryoprotectants dimethyl sulphoxide (DMSO) and glycerol,was investigated. The study also provided the opportunity to investigate the effect of developmental stage, with the associated changes in yolk content and cell size, on recovery following cryopreservation.

## Materials and Methods

### Ethics Statement

Collection of spawn and larvae of *Limnodynastes peronii* were approved under NSW NPWS scientific licence S10382 and the research protocol 706 0607 was approved by the University of Newcastle Animal Care and Ethics Committee.

### Collection of embryos

Spawn of Striped Marsh Frogs (*Limnodynastes peronii*) were collected from the wild or from animals spawning in captivity. *L. peronii* is a foam-nesting species that deposits its fertilised eggs into a floating foam nest produced from oviducal secretions that the female beats into a froth as eggs are deposited in pond water during amplexus.

**Figure 1:**
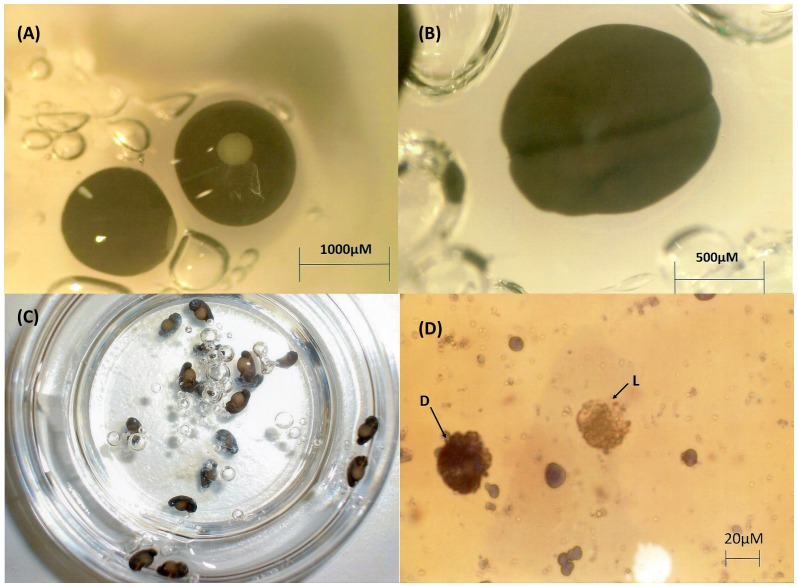
*Limnodynastes peronii* embryos and embryonic cells. (**A**) Late gastrula (Gosner Stage 12). (**B**) Neurula (Gosner Stage 15). (**C**) *L. peronii* embryos continuing normal development 24 hours after removal from holding at 8°C. (**D**) Post thaw viable and non-viable neurula cells determined with trypan blue live (L) - dead (D) staining; 400× magnification.

*L. peronii* spawn were held in disposable plastic containers with a small volume of pond water in an incubator at 8°C until required, but for no longer than 5 days (temperature/interval for which the embryos could be held without adverse effects, whilst holding development at desired stages). Embryos were used in experiments at two developmental stages [38]: mid to late gastrula (Gosner stages 10–12) or neurula (Gosner stages 14–16); Fig. 1a, 1b.

### Preparation of embryos for experimental protocols

Embryos were removed from refrigeration and allowed to equilibrate at room temperature 30 minutes prior to dissociation. During each experiment, some embryos were withheld and maintained at room temperature in petri dishes overnight to verify viability of embryos after the holding period (Fig. 1c).

A stereoscopic microscope (Model SZH-ILLB, Olympus) was used on low magnification with an external fibre optic light source to view embryos for determining stage of development and removing egg jelly. The outer, sticky jelly layer was removed for all experimental procedures. This was performed under the stereoscopic microscope whilst resting the embryo on a sheet of plastic coated absorbent paper (Benchkote, Whatman). The embryo was gently rolled on the absorbent paper to remove residual nest mucous “foam”, before the egg jelly layers were removed with fine forceps, leaving the embryo encapsulated within the vitelline membrane.

### Dissociation of Embryos

Individual de-jellied embryos were transferred into droplets on the surface of plastic petri dishes containing 25 µL Ca^2+^-free Simplified Amphibian Ringer (SAR) with 4 mM ethylenediaminetetraacetic acid (EDTA, Sigma) (referred to herein as Ca^2+^-free SAR/EDTA; see below). The droplets containing the embryos were left for 15 mins after which a further 10 µL of the same solution was added, and a 0.5–250 µL yellow pipette tip (Finntip 250 µL universal, Thermo Fisher Scientific) with ∼1.5 mm cut off the tip was used to transfer the embryos (within the ∼35 µL droplets) into 1500 µL Eppendorf tubes. A gentle up and down pipetting action was used to dissociate the embryos within the Eppendorf tube. The resulting cell suspensions were left for a further 10 mins prior to being used in experimental protocols.

### Solutions for dissociating and culturing embryos

The following solutions were used to dissociate and culture embryos in experiments for up to 24 hours: Simplified Amphibian Ringer (containing calcium) consisting of 113 mM NaCl, 2 mM KCl, 1.35 mM CaCl_2_,1.2 mM NaHCO_3_ [39], [40]; calcium-free SAR with 4 mM EDTA (Ca^2+^-free SAR/EDTA); culture medium (CM) consisting of 0.67× Dulbecco's Modified Eagle's Medium (Gibco) v/v, 10 mM HEPES, 3 mM NaHCO_3_, 0.1% w/v bovine serum albumin (BSA, CSL), 2% v/v Penicillin-Streptomycin (Sigma), 0.8% v/v Fungizone (Thermo Scientific), and 5 µL/100 ml Tween 80 (Sigma).

### Culturing and assessing dissociated embryos

The effect of culture in various solutions on viability of dissociated embryos over 24 hours was investigated by diluting the dissociated embryos (described above) in 3 types of solution: Ca^2+^-free SAR/EDTA, SAR or CM. As an additional treatment to test the effect of centrifugation on dissociated cell viability, some dissociated embryos were centrifuged at 400×g for 3 minutes, and resuspended in CM. Each treatment was replicated in N = 6 embryos (2 each from 3 different spawns).

All 24 hour incubation solutions were added stepwise (total of 250 µl) to the ∼35 µL starting volumes to minimise osmotic disruption as follows: 0 min, 5 µl; 5 min, 15 µl, 10 min, 30 µl, 15 min, 200 µl. Tubes were incubated at room temperature. 20 µl sub-samples were removed at 0.5, 1.5, 2.5 and 24 hours to assess the proportion of viable cells by the addition of 10 µl of 0.4% w/v trypan blue (Sigma). Cells were examined in the chamber of an improved Neubauer haemocytometer under an Olympus BH-2 compound microscope at 400× magnification and at least 100 cells were counted in each replicate; cells excluding the trypan blue dye (Fig. 1d) were scored as viable; cells taking the dye were scored as non-viable. The use of trypan blue to assess cell viability was tested against an alternative fluorescent staining technique (propidium iodide, SYBR® 14, Invitrogen, Oregon, USA; [41]) with no significant difference in results (data not shown).

### Cryoprotectant Toxicity

The cytotoxicity of the cryoprotectants dimethylsulphoxide (DMSO, Ajax Chemicals) and glycerol (BDH Chemicals) to cells at room temperature were tested by the stepwise addition of 250 µl of SAR or CM containing 5, 10, 15 or 20% v/v of cryoprotectant to individual embryos dissociated (as described previously) in 35 µl of Ca^2+^ free SAR. The 250 µl cryoprotectant solutions (or SAR or CM controls with no cryoprotectant) were added stepwise to the 35 µl of dissociated embryo in Eppendorf tubes as 5 µl at t = 0 mins, 15 µl at 10 mins, 30 µl at 15 mins, 200 µl at 20 mins and these were inverted at the time of addition to mix solutions; aliquots were removed to assess the proportion of viable cells using trypan blue (as described above) at 30 mins (after the first of the stepwise additions of cryoprotectant), and at 1.5, 2.5 and 24 hours after the first addition of cryoprotectant to each dissociated embryo. Each treatment (and control) was replicated in 6 embryos (2 embryos from each of 3 spawn).

### Cryopreservation of cells from dissociated embryos

The same cryoprotectant/media configurations described above for cryoprotectant toxicity tests were used in a cryopreservation experiment to determine the rate of post-thaw recovery of viable cells following cryopreservation. 150 µL of dissociated cell preparations (prepared as for the cryoprotectant tolerance experiment) were loaded into 250 µL semen straws (IMV technologies, France) and sealed with poly vinyl alcohol powder. Samples were cooled and frozen in a controlled cryochamber (Cryologic, Melbourne, Australia). The following cooling protocol was applied: straws prepared from samples held at room temperature were equilibrated at 10°C for 10 minutes, cooled to −7°C at a rate of −1°C/min, held at −7°C for 10 mins, cooled at −1°C/min to −30°C, held at −30°C for 10 mins before being allowed to go into free fall. When the temperature of the chamber was <−100°C, the straws were removed and plunged into liquid nitrogen where they were subsequently stored. The effect of seeding of straws at −7°C (using forceps cooled in liquid nitrogen) showed no effect on recovery rates (data not shown), and was not used in the cooling protocol.

Straws were thawed in air at room temperature and equilibrated for 30 minutes. The contents were emptied into 1.5 mL Eppendorf tubes, and 10 µL aliquots were stained with trypan blue to determine viability as previously described. Counts were performed at 0.5, 1.5, 2.5 and 24 hours post thaw; each treatment was replicated in 6–10 embryos. Due to low recovery of viable cells, the number of cells counted in replicates was increased wherever possible, with more than 500 cells counted in 56% of replicates (up to 1020 in gastrulae, 2400 in neurulae).

### Analyses of Data

Data for embryonic cell culture and cryoprotectant cytotoxicity experiments were converted to percentages, arcsine transformed, and subjected to repeated measures analysis of variance using Statistica 5.5 with media, cryoprotectant type, cryoprotectant concentration and embryonic stage as the other main effects. *Post hoc* tests (Duncan's multiple range and Planned Contrasts within the ANOVA routine in Statistica 5.5) were used to test hypotheses of specific differences between groups of means, and for quadratic effects within the cryoprotectant toxicity data. Due to the high number of zero values and the data following a poisson distribution, the cryopreservation data were analysed using the log likelihood function within the Statistica 5.5 Visual GLZ procedure.

## Results

### Dissociation of embryos and culture of embryonic cells

**Figure 2:**
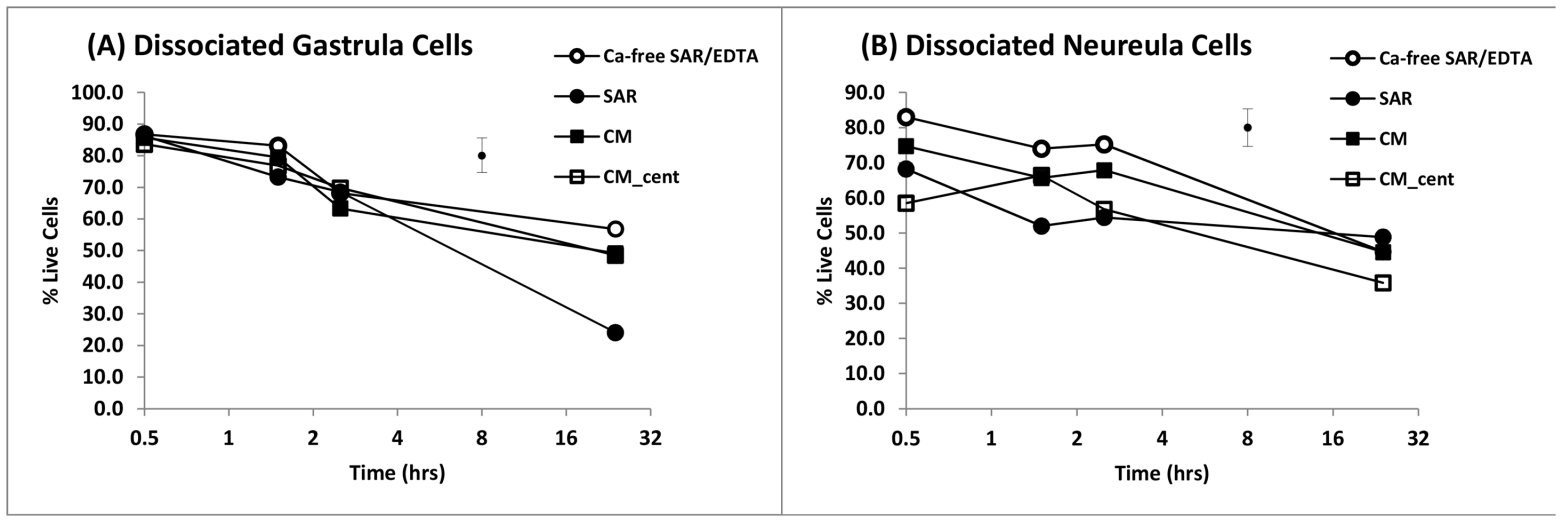
Mean viability of cells from gastrulae and neurulae dissociated in Ca^2+^-free SAR/EDTA and incubated in various media for 24 hours. (**A**) gastrulae; (**B**) neurulae. Ca^2+^-free-SAR/EDTA = Ca^2+^-free-SAR containing 4 mM EDTA; SAR = Simplified Amphibian Ringer; CM = culture medium; CM_cent = centrifuged and resuspended in CM. Each mean, n = 6 (2 embryos from each of 3 spawns); error bar = pooled standard error.

The viability of cells recovered from dissociated embryos was initially high for both gastrulae and neurulae, but declined in all media over 24 hours. The mean viability of dissociated cells taken from gastrulae (Fig. 2a) were high and within a narrow range (range of means 83.4 to 86.7% at 0.5 hr, the highest being in Ca^2+-^free SAR/EDTA); mean viability for neurula cells at 0.5 hr was also highest in Ca^2+-^free SAR/EDTA (83.0%, Fig. 2b), but ranged lower in other media (58.5 to 74.8%).

There were significant effects of embryonic stage (gastrula versus neurula; p<0.01), media (p<0.05) and time (p<0.001) on the proportion of viable cells following dissociation, as well as significant interactions between embryonic stage and time (p<0.001). The data indicated that the recovery of viable cells after dissociation was generally higher in gastrulae than neurulae, but not in all equivalent treatments at equivalent times; in cells of both embryonic stages there was a significant decline over 24 hour of culture (Fig. 2).

There was a benefit to cells dissociated and maintained in Ca^2+-^free SAR with EDTA compared to SAR containing calcium (p<0.02), or to culture media (p<0.05) which also contained calcium. The significant differences between media were largely due to differences between means from neurulae (SAR/EDTA 83.0±2.1%, SAR 68.2±3.3%, CM 74.8±2.1% at t = 0.5 hr; p<0.05; Fig. 2b); within the gastrulae (Fig. 2a), the media were not significantly different (e.g. SAR/EDTA 86.7±4.4%, SAR 85.7±3.7%, CM 83.6±3.9% at t = 0.5 hr) except for SAR at t = 24 hrs, which was significantly lower (p<0.01) than the other media.

Viability of dissociated cells was not significantly reduced as a result of concentrating cells by centrifugation and resuspending in CM for either gastrulae (p = 0.967) or neurulae (p = 0.135).

### Cryoprotectant Toxicity (unfrozen cells)

**Figure 3:**
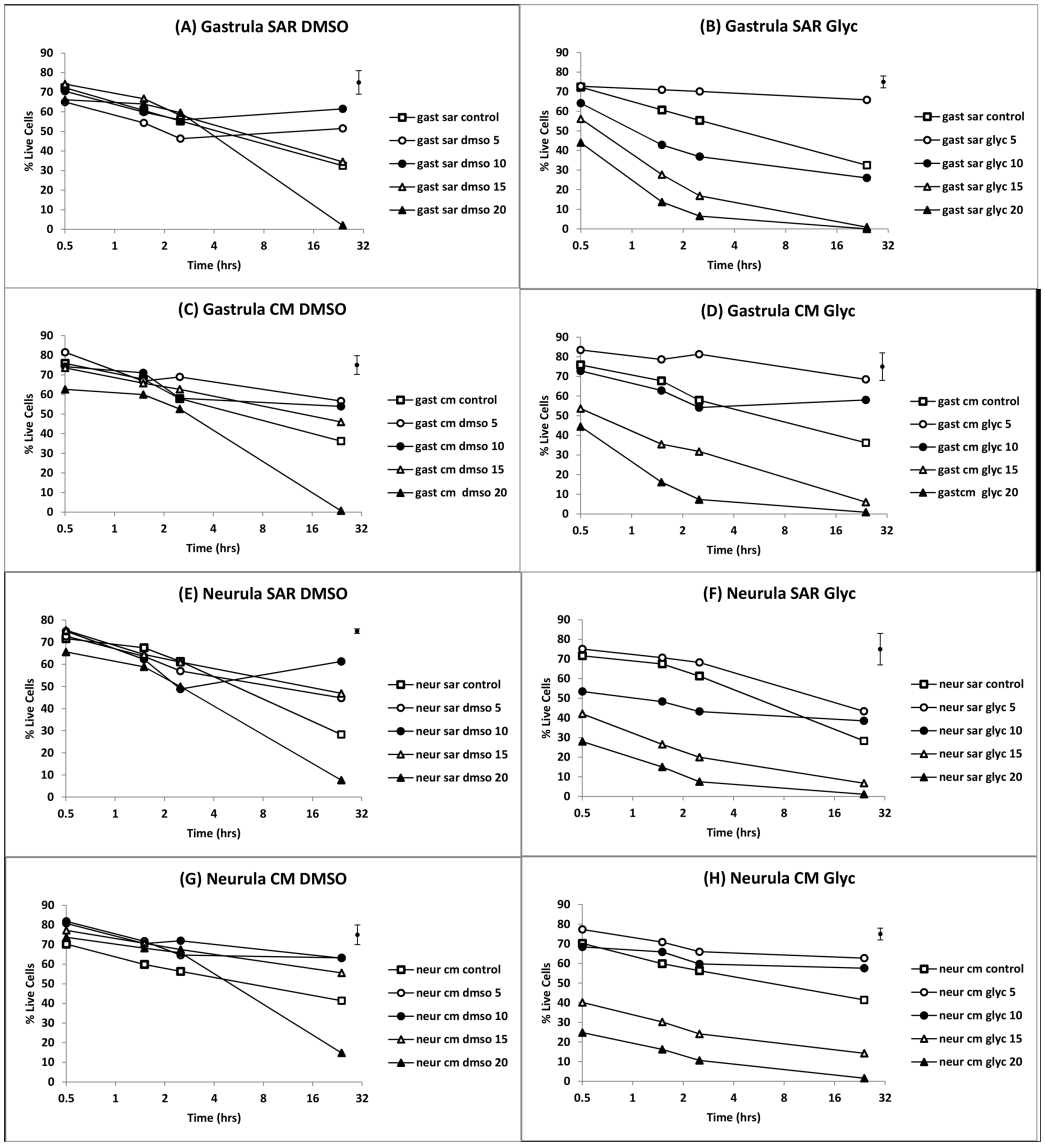
Mean viability of cells from gastrulae and neurulae incubated in various media/cryoprotectant combinations for 24 hours. (**A–D**) gastrulae; (**E–H**) neurulae. CM = culture medium; SAR = Simplified Amphibian Ringer; dmso = DMSO; glyc = glycerol; numbers 5–20 indicate % v/v concentration of cryoprotectant in media; control = 0% v/v cryoprotectant. Each mean, n = 6 (2 embryos from each of 3 spawns); error bar = pooled standard error.

**Figure 4:**
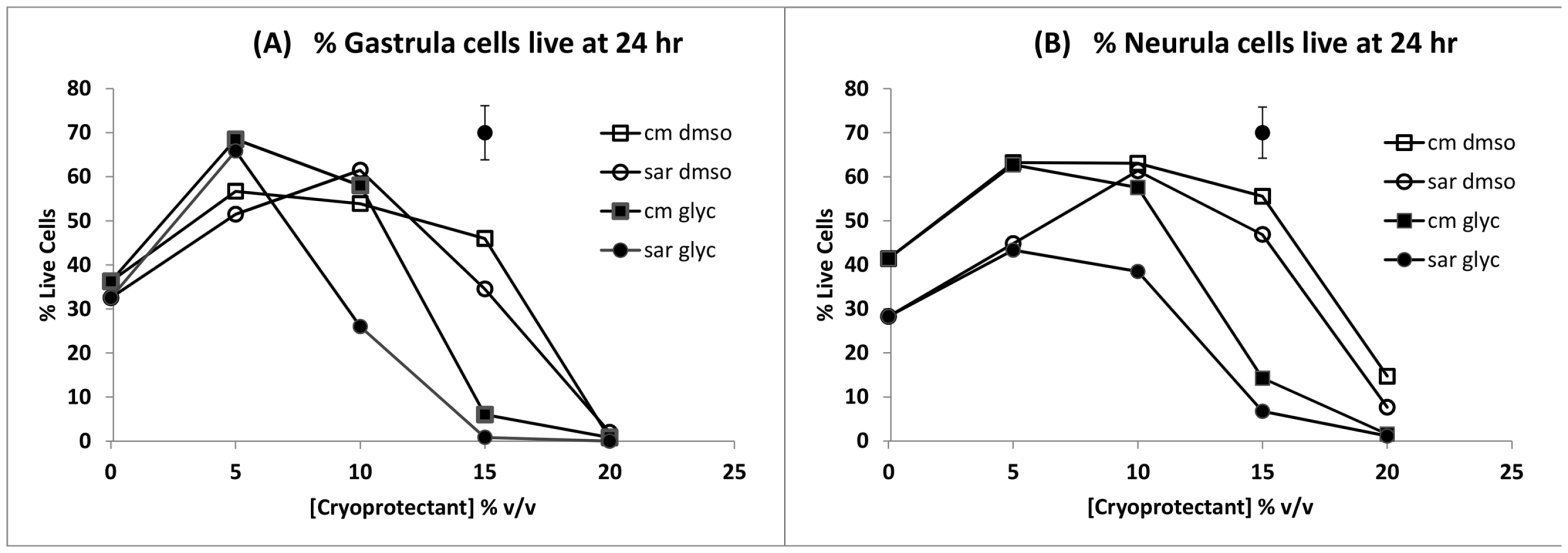
Mean viability of cells from gastrulae and neurulae after 24 hours of incubation in various media/cryoprotectant combinations. (**A**) gastrulae; (**B**) neurulae. cm dmso = CM containing DMSO, sar dmso = SAR containing DMSO, cm glyc = CM containing glycerol, sar glyc = SAR containing glycerol. Each mean, n = 6 (2 embryos from each of 3 spawns); error bar = pooled standard error.

The viability of gastrula and neurula cells from dissociated embryos cultured for 24 hours in CM or SAR in the presence of DMSO or glycerol (Figs. 3 and 4) showed significant main effects for time, media, cryoprotectant type and cryoprotectant concentration (all p<0.001) but no significant main effect of embryonic stage (gastrula versus neurula, p = 0.416). There were also significant interaction effects including cryoprotectant type×cryoprotectant concentration (p<0.01), cryoprotectant type×time (p<0.01) cryoprotectant concentration×time (p<0.001), and some higher order effects. The lack of a significant main effect for embryonic stage was indicative of a similar overall response of gastrula and neurula cells to cryoprotectant and other treatments (visible by inspection of Fig. 3), although there were some significant higher order interaction effects involving embryonic stage e.g. stage×media×concentration×time (p<0.01).

The presence of cryoprotectants in the media over 24 hours of incubation had complex effects on the viability of cells related both to cryoprotectant type and concentration (Figs. 3 and 4), as indicated by the significant interaction effects described above. These included higher survival of cells in media containing cryoprotectants at low concentrations than in cryoprotectant free controls (p<0.05), but lower survival at higher cryoprotectant concentrations (p<0.001). DMSO and glycerol both significantly (p<0.05) improved viability over controls at low concentrations. The significantly higher overall viability (main effect; p<0.001) of cells in DMSO than glycerol was due to the toxicity of glycerol at high concentrations, rather than at low concentrations where it was generally no more toxic, or in some cases, less toxic than DMSO.

Overall, trends were similar in SAR and CM media. However, beneficial effects of cryoprotectant on viability were maintained to higher concentrations in CM than SAR (p<0.001), an effect that was most obvious after 24 hours of incubation (Fig. 4). SAR/glycerol were the most toxic media/cryoprotectant combinations at higher concentrations after 24 hours, indicating that SAR was less effective than CM at reducing toxic effects of higher concentrations of glycerol (Fig. 4).

Beneficial effects of cryoprotectants on survival after 24 hours resulted in high mean 24 hr values for many of the 5% and 10% v/v treatments compared to the cryoprotectant free controls e.g. in gastrula cells: 68.5±2.9% in CM/5% glycerol and 56.7±8.2% in CM/5% DMSO versus 36.2±3.4% in CM control; in neurula cells: 62.7±4.2%, CM/5% glycerol and 63.2±4.3% in CM/5% DMSO versus 41.4±4.3% in CM controls. The beneficial effect of 5–10% v/v cryoprotectant concentrations on viability in comparison to media with no cryoprotectant is evident in Figure 4, where there was a statistically significant quadratic effect of concentration on viability at 24 hours of incubation (p<0.001) due to viability rising significantly with cryoprotectant concentration initially before declining at higher concentrations. For gastrulae (Fig. 4a), 5 and 10% w/v glycerol and DMSO means were higher than cryoprotectant free controls with the exception of 10% w/v glycerol in SAR. For neurula cells (Fig. 4b), all 5 and 10% w/v cryoprotectant concentrations were equal to or greater than controls; and 15% w/v DMSO means were higher than controls as well.

### Recovery of gastrula and neurula cells after cryopreservation

Recovery of viable cells following cryopreservation was low in all treatments for both gastrula (Table 1) and neurula cells (Table 2). As an indicator of the low rates of recovery post-thaw, across all treatments and replicates, only 22% of gastrula replicates and 32% of neurula replicates recovered more than 1% viable cells, and only 7.5% of gastrula and neurula replicates had greater than 2% recovery. The highest cell recovery in any gastrula replicate was 4.4% (CM/10% DMSO) and in any neurula replicate was 5.1% (CM/15% glycerol) assessed at 0.5 hours post thaw. The highest mean recovery for gastrula cells was 1.1±0.8 (n = 6) in CM/10% DMSO and for neurula cells was 1.9%±0.7% (n = 8) in CM/20% glycerol.

**Table 1:** Recovery of gastrula cells after cryopreservation.

		Time (hours)							
		0.5		1.5		2.5		24	
Treatment	[CP] %v/v	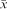	# Viable	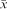	# Viable	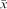	# Viable	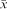	# Viable
CM control	0	0.0	0/5	0.0	0/5	0.0	0/5	0.0	0/5
CM/DMSO	5	0.0	0/6	0.6	1/6	1.4	1/6	0.0	0/6
	10	11.1	2/6	0.0	0/6	3.7	1/6	0.0	0/6
	15	8.9	4/6	0.5	1/6	3.1	2/6	0.0	0/6
	20	1.6	1/6	0.7	1/6	0.0	0/6	0.0	0/7
CM/Glycerol	5	1.7	1/6	0.0	0/6	1.2	2/6	0.0	0/6
	10	5.2	2/5	2.6	2/6	0.0	0/6	0.0	0/6
	15	6.4	3/5	0.0	0/6	1.5	1/6	0.0	0/6
	20	9.8	4/6	1.9	2/6	1.5	2/6	0.0	0/6
SAR control	0	0.0	0/6	0.0	0/6	0.0	0/6	0.0	0/6
SAR/DMSO	5	3.5	2/6	0.0	0/6	0.0	0/6	0.0	0/5
	10	8.3	3/6	1.7	1/6	0.0	0/6	0.0	0/6
	15	1.4	1/6	1.8	1/6	0.0	0/6	0.0	0/6
	20	0.0	0/6	0.0	0/6	0.0	0/6	0.0	0/6
SAR/Glycerol	5	5.9	2/6	2.1	2/6	3.5	2/6	0.0	0/6
	10	9.9	5/6	3.9	4/6	4.3	3/6	0.0	0/6
	15	2.7	1/6	5.0	1/6	4.4	3/6	0.0	0/6
	20	6.5	2/6	8.0	2/6	7.4	2/6	0.0	0/6

Post-thaw recovery of cell viability (live/dead staining) expressed as mean number of viable cells per thousand counted (left hand column at each time) and the number of replicates with viable cells (right hand column at each time) for each treatment group. [CP] = cryoprotectant concentration.

**Table 2:** Recovery of neurula cells after cryopreservation.

		Time							
		0.5		1.5		2.5		24	
Treatment	[CP] %v/v	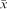	# Viable	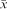	# Viable	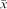	# Viable	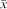	# Viable
CM control	0	0.0	0/8	0.0	0/8	0.0	0/8	0.0	0/8
CM/DMSO	5	2.8	5/13	2.3	5/13	0.0	0/13	0.0	0/13
	10	4.8	6/10	2.3	4/10	0.3	1/10	0.2	1/10
	15	3.6	8/10	1.4	5/10	0.0	0/10	0.2	1/10
	20	6.8	11/14	1.4	8/14	0.2	1/14	0.0	0/14
CM/Glycerol	5	2.2	3/8	1.0	3/8	1.0	2/8	0.3	1/8
	10	9.3	6/8	5.7	6/8	4.0	4/8	0.0	0/8
	15	17.2	7/8	8.9	7/8	6.1	7/8	0.6	2/8
	20	19.1	7/8	6.5	7/8	4.9	8/8	0.5	1/8
SAR control	0	0.0	0/8	0.0	0/8	0.0	0/8	0.0	0/8
SAR/DMSO	5	2.0	4/8	0.3	1/8	0.0	0/8	0.0	0/8
	10	5.8	8/8	1.2	4/8	1.2	4/8	0.0	0/8
	15	4.9	6/8	1.8	3/8	0.6	3/8	0.0	0/8
	20	5.8	6/8	2.7	4/8	1.2	3/8	0.0	0/8
SAR/Glycerol	5	2.7	5/8	2.0	4/8	1.5	1/8	0.5	1/8
	10	8.7	8/8	3.9	6/8	2.4	5/8	0.0	0/8
	15	11.4	8/8	6.0	6/8	3.2	5/8	0.4	1/8
	20	12.7	7/8	4.8	5/8	3.2	4/8	0.3	2/8

Post-thaw recovery of cell viability (live/dead staining) expressed as mean number of viable cells per thousand counted (left hand column at each time) and the number of replicates with viable cells (right hand column at each time) for each treatment group. [CP] = cryoprotectant concentration.

When all data at 0.5 hours post thaw were analysed together, the mean recovery of viable cells was higher in neurula (6.5 cells per thousand; cpt) than gastrula cells (4.6 cpt), p<0.001; in CM (6.2 cpt) than SAR (5.3 cpt), p<0.01; in glycerol (8.2 cpt) than DMSO (4.5 cpt, p<0.001) and there was a significant mean increase in recovery with increasing concentration of cryoprotectant ( 2.6, 5% v/v; 7.7 cpt, 10% v/v, 7.3 cpt, 15% v/v, 8.1, 20% v/v; p<0.001). The higher recovery of cryopreserved cells from neurulae at 0.5 hours persisted with time, and only thawed neurula cells were still viable after 24 hours of incubation (Table 2).

Nevertheless, there were major, significant interaction effects in mainly the gastrula, but also the neurula data that complicate simple identification of the most effective cryopreservation protocols. These included significant media×cryoprotectant type (p<0.01), media×cryoprotectant concentration (p<0.001), cryoprotectant type×cryoprotectant concentration (p<0.001) and even media×cryoprotectant type×cryoprotectant concentration (p<0.001) effects for gastrula cells. Cryoprotectant (glycerol 6.0 cpt vs DMSO 4.4 cpt) and cryoprotectant concentration, but not media, main effects were significant (p<0.01) in gastrula cells. For neurula cells, interaction effects weren't as strong with only cryoprotectant type×cryoprotectant concentration interactions significant (p<0.001), while cryoprotectant (glycerol 10.4 cpt vs DMSO 4.6 cpt) and cryoprotectant concentration (p<0.001), but not media, main effects were significant.

Notwithstanding the complexities of the interactions, reasonable interpretations of the data in Tables 1 and 2 lead to the conclusions that: (1) recovery of cells from cryopreservation is higher and persists longer in neurula cells; (2) media effects on recovery are weak or only apparent as complex interaction effects; (3) recovery is higher overall in glycerol than in DMSO, but DMSO recovery can be comparable in favourable media/cryoprotectant concentration combinations; (4) recovery in both glycerol and DMSO is generally higher in concentrations above 5% v/v. These data allow the identification of 10–15% DMSO/CM and 10–15% glycerol/SAR as favourable cryodiluents for gastrula cells and 15–20% glycerol in CM or SAR as favourable cryodiluents for neurula cells (taking into account the persistence of viability at 2.5 hours post thaw).

## Discussion

This study found that cells from dissociated embryos of *L. peronii* can be cryopreserved by slow cooling, although the rate of recovery of viable cells, as assessed by live/dead staining, is very low. The optimal cryodiluents identified in this study were 10–15% DMSO/CM and 10–15% glycerol/SAR for gastrula cells and 15–20% glycerol in CM or SAR as favourable cryodiluents for neurula cells. There are no other reports of amphibian embryonic cell cryopreservation against which to benchmark this study with the exception of the report of Uteshev and colleagues [36], [42], in which cells from blastulas of *Rana temporaria* were vitrified in a cryoprotectant medium containing 10% sucrose and 10% DMSO. In that study, a high recovery rate of 87% of cryopreserved blastomeres was reported. The reasons for such a large difference in recovery rates between the two studies will require further investigation but may be due to many factors including species differences, embryonic stage (blastula versus gastrula/neurula), handling procedures, cryodiluent composition and cooling rate (slow cooling versus vitrification). The availability of only two reports on amphibian embryonic cell cryopreservation leaves many questions unanswered. In this study, the viability of unfrozen cells, even in the presence of high levels of cryoprotectants, for up to 24 hours indicates that the cryopreservation process, not the handling procedures were the cause of the loss of viability.

Unfrozen cells of *L. peronii* embryos were shown to have a high tolerance to cryoprotectants at room temperature, and even to benefit from their presence in the media over extended periods of incubation (with many combinations of cryoprotectant and medium maintaining above 60% viability after 24 hours). Interestingly, cell viability was maintained at a higher level in the presence of low to medium levels of penetrating cryoprotectant (5–10% v/v DMSO and glycerol) than in cryoprotectant free controls, with increases of more than 20–25% in survival at 24 hours of incubation. Determining the reasons for the beneficial effect of cryoprotectant in the media were beyond the scope of this study, but may derive from a wide range of mechanisms such as anti-oxidant or membrane stabilising effects. Comparable studies on isolated fish blastomeres have also reported high levels of tolerance to DMSO including rainbow trout (85% intact in 8.7% v/v DMSO; [26]), chum salmon (90% survival after 30 mins in 5 or 10% v/v DMSO, and 65% survival in 20% v/v DMSO; [24]), zebrafish (no reduction in hatching of embryos after 2 hours in 7% v/v DMSO; [23]) and medaka, whiting and pejerrey (greater than 80–90% survival after more than 5 hours in up to 9% v/v DMSO [43]). Higher concentrations above those described were associated with significant toxic effects in a number of these species. In contrast, much lower levels of glycerol have been reported as toxic to fish blastomeres: [24] reported only 65% survival in 5% v/v glycerol, and less than 20% in 10% v/v, and less than 10% in 20% v/v glycerol in chum salmon blastomeres following 30 minutes of glycerol exposure; [44] abandoned attempts to investigate permeability of zebrafish embryos to glycerol because of its toxicity to early embryos. None of those fish studies reported the increase in survival found with low concentrations of cryoprotectant reported here for *L. peronii* embryonic cells. The data indicate that there is no need to remove cryoprotectants from media containing *L. peronii* embryonic cells, and that these cryoprotectants may even be retained in media for purposes of cell stabilisation. It also worth noting that combinations of cryoprotectants and culture medium generally supported cell viability at a higher level than cryoprotectants with Ringer.

The data from this study suggest that dissociated cells benefited from a low calcium environment (at least, in the absence of cryoprotectants in the media) since survival over 24 hours was higher in Ca^2+^ free SAR than SAR with Ca^2+^. This effect was only significant in neurula cells. Ca^2+^-free media was used to dissociate embryos in this study, as in other studies involving fish and frog embryos [19], [30], [45] as this reduces damage to cells from mechanical disruption of cell to cell junctions (Ca^2+^ is required to maintain intercellular junctions and cell adhesion, [46]–[48]). However, since all replicates were first dissociated in Ca^2+^ free media, the lower viability of neurula cells in Ca^2+^ -SAR cannot be attributed to the presence of Ca^2+^ during dissociation, but must be due to other effects post-dissociation when Ca^2+^ was added back. Interpretation of these data may be complicated by the observation of re-aggregation of neurula cells in some replicates in the presence of Ca^2+^ (data not shown), which may have increased the relative proportion of non-viable cells in suspension by selective re-aggregation of viable cells.

The recovery of post thaw viability of cryopreserved cells did not follow the same pattern of response to cryoprotectant concentration as the unfrozen cells. In unfrozen cells, viability was significantly decreased by higher concentrations, but in the cryopreservation treatments, there was a trend for higher recovery rates in higher concentrations of glycerol and DMSO, although the effects were complicated by complex interactions. It would appear that the protective effects of higher cryoprotectant concentrations during cryopreservation outweigh the toxic effects during incubation at room temperature. The recovery of cryopreserved *L. peronii* cells following cryopreservation was low compared to reports of the recovery of fish blastomeres after cryopreservation; in this study mean recovery rates were less than 2% with the highest recovery in a gastrula replicate 4.4%, and neurula replicate 5.1%. Most reports of fish blastomere cryopreservation are for cryodiluents employing DMSO. Depending on the species, and the method of freezing, the results are variable, but in all cases higher than values for *L. peronii*: zebrafish 70–85% recovery by slow cooling in 7–10% v/v DMSO [23], [25] and 93% with vitrification in 35% v/v DMSO [21]; rainbow trout 36% recovery in 8.7% DMSO [26], up to 95% recovery in 1.4 M propanediol [19]; chum salmon 59% recovery in 10% v/v DMSO/10% [24]; pejerrey 67% recovery in 18% DMSO, but lower recovery rates for whiting (20%) and medaka (34%) in 9% v/v DMSO [43]. Nevertheless, even with fish blastomeres, large variations in recovery rates occur with variations in cryoprotectant type and concentration [24], [43], and some treatments yield recovery rates as low as values found in this study. It is difficult to make meaningful comment on differences in recovery rates between fish and amphibian embryonic cells, given this is the only study of cryopreservation by slow cooling with no comparable data from other studies of amphibians, and the only other report by [36] involving vitrification. Factors that may contribute to the differences include the developmental stage (most fish studies involve blastomeres from 50% epiboly blastula versus gastrula and neurula in this study), methodological differences, and potentially fundamental differences between fish and amphibians in the response to cryopreservation. The yolk content of amphibian oocytes and embryos may be even higher than that of fish in some cases (compare [18], [49] to values from [44]).

One response that was similar between this study and studies reporting the cryopreservation of fish blastomeres was the trend for recovery rates to be higher in later stages of embryo development in fish (across varying stages of blastula [19], [43], and in this study higher recovery of neurula than gastrula cells). This may be due to many factors, but could include more favourable biophysical properties of the smaller cells generated as cleavage proceeds in pre-hatching embryos [45], [50]. In the current study, gastrula cells of *L. peronii* were mostly in the order of 30–70 µm and neurula cells in the range of 20–30 µm. [14] also reports a number of fish species in which chill sensitivity in whole embryos declines as embryonic development proceeds, paralleling the results with blastomere cryopreservation.

It should be recognised that even though the rate of recovery of intact cryopreserved *L. peronii* embryonic cells was low, this may not indicate that use of cryopreserved cells will not be successful in the generation of nuclear transfer embryos. Although cell viability assessed by the recovery of intact cells with sufficient membrane integrity to exclude vital stains was the measure of response in this study, the integrity of the cell nuclei may still be high, and they may be capable of supporting nuclear transfer. In amphibian nuclear transfer (although not the case with chimeras), cell membranes are deliberately disrupted when cell nuclei are transferred [37].

This study is a step forward in investigating the cryopreservation of amphibian embryonic cells, but poses many questions that require further investigation if cryopreserved embryonic cells are to generate viable embryos for the use in amphibian genome banking and species management for conservation. Further studies need to investigate whether recovery can be boosted by changing factors such as cooling and thawing rates, and cryodiluent composition e.g. improvements in fish blastomere cryopreservation by including foetal calf serum as reported by [24].
